# Metalloproteinases (MMPs) in hypertensive disorders: role, function, pharmacology, and potential strategies to mitigate pathophysiological changes

**DOI:** 10.3389/fphar.2025.1559288

**Published:** 2025-05-26

**Authors:** Soroush Taherkhani, Mohammad Sheibani, Ali Mohammadkhanizadeh, Jitka A. I. Virag, Lisandra de Castro Braz, Yaser Azizi

**Affiliations:** ^1^ Department of Physiology, School of Medicine, Iran University of Medical Sciences, Tehran, Iran; ^2^ Razi Drug Research Center, School of Medicine, Iran University of Medical Sciences, Tehran, Iran; ^3^ Department of Pharmacology, School of Medicine, Iran University of Medical Sciences, Tehran, Iran; ^4^ Department of Physiology, East Carolina University, Greenville, NC, United States

**Keywords:** MMPs, ECM, hypertensive disorders, vascular remodeling, TIMPs

## Abstract

Matrix metalloproteinases (MMPs) are a family of enzymes that play an important role in the pathophysiology of hypertensive disorders, particularly through their involvement in extracellular matrix (ECM) remodeling and vascular dysfunction. Their activity is closely linked to hypertension-mediated organ damage, which affects the vascular and cardio-renal systems. MMPs are responsible for degrading various components of the ECM, which is crucial for maintaining vascular structure and function. In hypertensive patients, several MMPs, including MMP-1, MMP-3, and MMP-9, are often found at elevated levels. This is associated with vascular remodeling and dysfunction due to chronic high blood pressure. The activation of MMPs in hypertension can be triggered by several factors, such as oxidative stress, inflammatory cytokines, and vasoactive agents like angiotensin II. In addition to increasing MMP activity, these variables cause an imbalance between MMPs and tissue inhibitors of metalloproteinases (TIMPs), which are the MMPs’ natural inhibitors. This imbalance contributes to excessive degradation of the ECM and promotes pathological changes in vascular smooth muscle cells (VSMCs), leading to their transition from a contractile to a synthetic phenotype. This shift facilitates cell growth and migration, exacerbating vascular remodeling. Given their critical roles in hypertension-related organ damage, MMPs are being explored as potential pharmacological targets. Inhibitors of MMPs may help mitigate the adverse effects of hypertension by restoring balance in ECM remodeling processes. Understanding their mechanisms opens avenues for targeted therapies that could significantly improve outcomes for individuals suffering from hypertension-related complications.

## Graphical Abstract

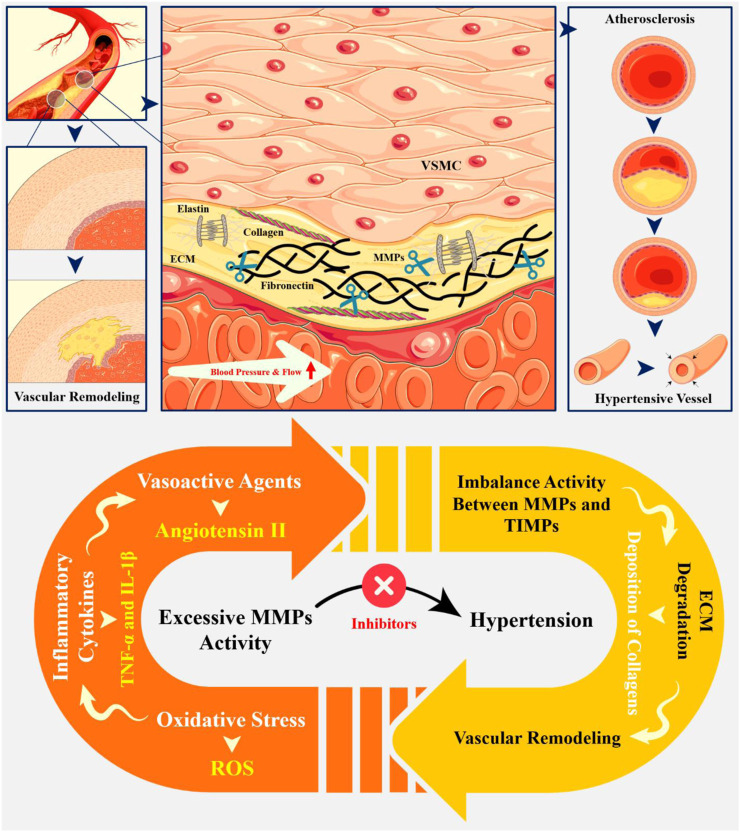

## Highlights

• In hypertensive conditions, increased MMP activity promotes vascular remodeling characterized by intimal-medial thickening, fibrosis, and calcification.
• MMPs are upregulated by various stimuli, including Angiotensin II and TNF-α, leading to increased ECM degradation and increased blood pressure.
• Utilizing specific MMP inhibitors to restore balance between MMPs and TIMPs may help prevent excessive ECM degradation and support vascular integrity.
• Implementing antioxidants can reduce oxidative stress levels that contribute to increased MMP activity.

## 1 Introduction

Hypertensive disorders, often referred to as high blood pressure or hypertension, represent a significant public health concern affecting millions of individuals around the globe. These conditions, which manifest through persistently elevated blood pressure lead to severe health complications if left untreated (Sutton et al., 2018). Hypertensive disorders are classified into several categories, including gestational hypertension, preeclampsia, chronic hypertension with superimposed preeclampsia, and chronic hypertension (Lowe et al., 2015). The implications of hypertensive disorders extend beyond mere elevated blood pressure; they can lead to serious health issues such as organ damage, cardiovascular complications, and adverse pregnancy outcomes (Wilkerson and Ogunbodede, 2019). For instance, preeclampsia is particularly concerning as it can progress to eclampsia, a life-threatening condition marked by seizures. Understanding the risk factors associated with hypertensive disorders is essential for prevention and effective management. Factors such as obesity, a history of hypertension, advanced maternal age, and certain genetic predispositions can increase the likelihood of developing pre-eclampsia (Magee et al., 2014).

Matrix Metalloproteinases (MMPs) are a group of zinc-dependent endopeptidases that play important roles in a variety of physiological and pathophysiological processes, including tissue remodeling, inflammation, and migration (Fontana et al., 2012). In the context of hypertensive disorders, MMPs have been shown to regulate blood pressure through their ability to degrade and process various vasoactive peptides, such as angiotensin II, bradykinin, and endothelin-1 (Androulakis et al., 2012). MMPs can modulate the activity of these peptides by cleaving them into active or inactive forms, thereby influencing blood pressure (Palei et al., 2008). For example, MMP-2 and MMP-9 have been shown to degrade big endothelin-1, a precursor of endothelin-1, into its active form, which is a potent vasoconstrictor (Palei et al., 2012a). This process can contribute to the development of hypertension. On the other hand, MMP-1 has been reported to degrade angiotensin II, a potent vasoconstrictor, into its inactive form, thereby reducing blood pressure (Marchesi et al., 2012).

Given the critical role of MMPs in the pathogenesis of hypertensive disorders, they have emerged as potential therapeutic targets for the treatment of hypertension and its associated cardiovascular complications (Palei et al., 2012b). Multiple MMP inhibitors have been developed, and preclinical research has indicated that they may lower blood pressure and prevent cardiovascular disease. As demonstrated in several studies, doxycycline has been shown to inhibit a number of MMPs, thereby contributing to an improvement in blood pressure and cardiac fibrosis. Mechanistically, doxycycline exerts its inhibitory effect on the activity of various MMPs, particularly MMP-2 and MMP-9, resulting in a reduction of extracellular matrix (ECM) degradation. This action is of particular significance in conditions where excessive MMP activity contributes to vascular pathologies, such as atherosclerosis, neointima formation after vascular injury, vascular remodeling, and intimal hyperplasia associated with hypertension (Liu et al., 2003; Pires et al., 2011; Lee et al., 2004; Ogut et al., 2016). Recent advancements in the development of selective MMP inhibitors highlight both progress and challenges in targeting specific MMP family members for therapeutic applications. There is a concerted effort to create new classes of MMP inhibitors with improved potency and selectivity for specific MMPs relevant to particular diseases. Current research focuses on developing selective inhibitors that avoid the broad-spectrum effects seen in earlier compounds, which often led to disappointing clinical outcomes, especially in cancer treatments. In this regard, recent studies have identified promising candidates, including selective MMP-1 inhibitors with very low IC50 values, suggesting high potency. Additionally, novel thiazole derivatives have been identified as potential anti-neoplastic agents by targeting MMPs, demonstrating selective inhibition profiles (Ogut et al., 2016; Nuti et al., 2007).

Moreover, there is ongoing research into allosteric inhibitors that do not rely on zinc-binding groups, aiming to minimize off-target effects associated with traditional MMP inhibitors that chelate zinc and other metals. Non-selective MMP inhibitors often affect multiple MMP family members, leading to unintended side effects and complicating therapeutic outcomes. These broad-spectrum inhibitors have generally failed in clinical trials due to adverse effects linked to their lack of specificity (Spinale and Villarreal, 2014). On the other hand, the lack of selectivity can result in the inhibition of beneficial MMP functions alongside harmful ones, complicating treatment strategies and highlighting the necessity for more refined approaches that target specific MMP family members without affecting others. The development of MMP inhibitors faces several significant challenges, particularly in the context of creating effective and selective therapeutic agents (Fischer and Riedl, 2021). Many existing MMP inhibitors are broad-spectrum, affecting multiple MMP family members and other zinc-dependent proteases. This non-selectivity can lead to undesirable side effects and complicates therapeutic outcomes. Despite extensive research and development efforts, no MMP inhibitor has successfully passed clinical trials (Nuti et al., 2007; Spinale and Villarreal, 2014; Fischer and Riedl, 2021). Also, MMPs share high structural homology, making it difficult to design inhibitors that selectively target specific MMP family members without affecting others. Moreover, many synthetic MMP inhibitors suffer from issues related to chemical stability and bioavailability, which can limit their effectiveness in clinical settings (Fischer and Riedl, 2021; Mohan et al., 2016).

While MMP inhibitors have been extensively studied, the role of MMP activators in modulating cardiovascular function is less understood. Activating specific MMPs may enhance their beneficial effects on ECM remodeling and vascular repair processes (Sakata et al., 2004). Several classes of drugs, including antihypertensive medications, have been found to influence MMP activity. For instance, nitroglycerin can increase expression and activity of MMP-2, MMP-7, and MMP-9 while decreasing TIMP-1 levels. Also, angiotensin-converting enzyme (ACE) inhibitors may increase MMP-1 activity, while losartan has been shown to elevate MMP-2 activity (Krishnatry et al., 2011).

By addressing these knowledge gaps, we can gain a deeper understanding of the role of MMPs in hypertensive disorders and develop effective therapeutic strategies for the treatment of hypertension and its associated cardiovascular complications.

## 2 Classification, characteristics and pathophysiology of hypertensive disorders

**FIGURE 1:**
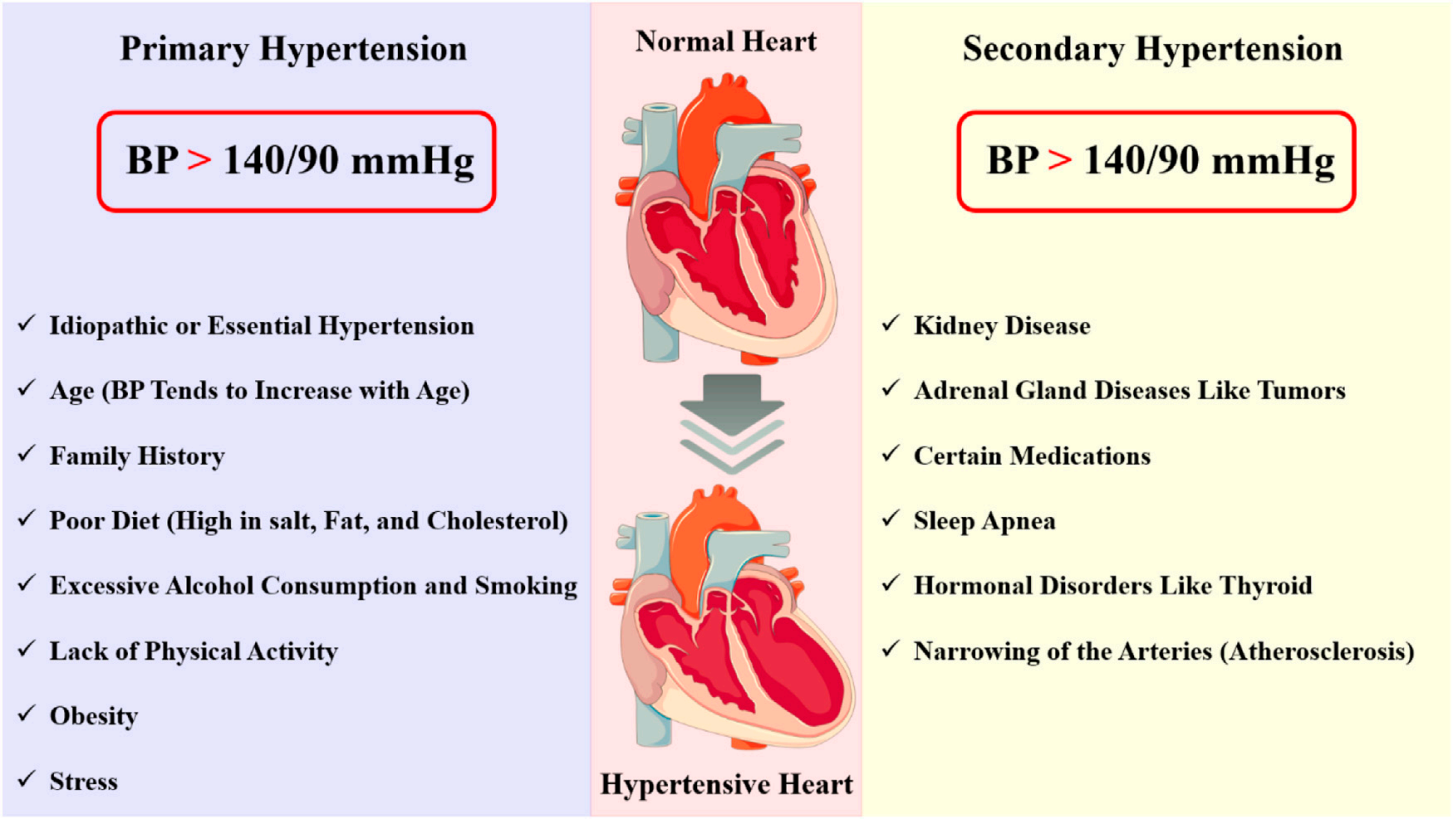
Hypertension is a complex disorder characterized by chronic elevation of blood pressure, which can lead to significant end-organ damage and increased morbidity and mortality. This disorder can be categorized into two types: primary (essential) hypertension and secondary hypertension. Each type presents distinct pathological changes and characterization.

Broadly classified as primary and secondary hypertension (Figure 1), the characteristics of hypertensive disorders are crucial for understanding their pathophysiology, diagnosis, and management. Primary hypertension, also known as essential hypertension, is the most common form of hypertension, accounting for approximately 90% of all cases. It is a multifactorial disorder, resulting from the interaction of genetic, environmental, and lifestyle factors and characterized by: (i) elevated blood pressure (BP) ≥140/90 mmHg; (ii) no identifiable cause or underlying condition; (iii) Gradual onset, often asymptomatic in the early stages; (iv) increased peripheral resistance and (v) normal or slightly decreased cardiac output (Luizon et al., 2014; Mistry and Pipkin, 2013). Secondary hypertension is caused by an underlying medical condition or factor that elevates blood pressure; It’s characterized by: (i) elevated BP ≥ 140/90 mmHg; (ii) identifiable underlying cause or condition, such as: kidney disease (e.g., chronic kidney disease and glomerulonephritis), adrenal gland disorders (e.g., Cushing’s syndrome and pheochromocytoma), thyroid disorders (e.g., hyperthyroidism), sleep apnea and medication-induced hypertension (e.g., non-steroidal anti-inflammatory drugs (NSAIDs) and birth control pills); (iii) often presents with symptoms, such as headaches, dizziness, and fatigue; and (iv) variable impact on cardiac output and peripheral resistance (Tranquilli et al., 2014; Ferrazzi et al., 2018).

In addition to primary and secondary hypertension, several subtypes of hypertensive disorders have been identified, including: (i) malignant hypertension: A rare, life-threatening condition characterized by severe hypertension (BP ≥ 200/120 mmHg) and rapid progression to end-organ damage; (ii) resistant hypertension: A condition in which BP remains elevated despite the use of three or more antihypertensive medications; (iii) isolated systolic hypertension: A type of hypertension characterized by elevated systolic BP (≥140 mmHg) and normal diastolic BP (<90 mmHg) and (iv) white coat hypertension: A condition in which BP is elevated only in a clinical setting, often due to anxiety or stress (Watanabe et al., 2017; Simonneau et al., 2004). Hypertensive emergency occurs when the blood pressure exceeds 180/120 mmHg, causing end-organ damage and dysfunction. The sudden and severe increase in blood pressure causes mechanical stress on the blood vessels, leading to endothelial dysfunction, inflammation, and vascular remodeling in conjunction with a cascade of signaling events that lead to vascular damage, inflammation, and oxidative stress. Blood pressure and vascular tone are mostly controlled by the endothelium, a single layer of cells that lines the blood vessels. In hypertensive emergency, the endothelium is damaged, leading to the release of vasoconstrictors, such as endothelin-1, and the reduction of vasodilators, such as nitric oxide. This imbalance favors vasoconstriction, further increasing blood pressure and exacerbating the condition (Talle et al., 2022; Simonneau et al., 2013).

The pathophysiology of hypertensive disorders involves a complex interplay of genetic, environmental (exposure to toxic metals, air pollution, climate, and noise), and lifestyle factors (such as dietary habits, physical activity, and stress) that contribute to the development and progression of hypertension (Simonneau et al., 2009). Familial clustering of hypertension has been observed, suggesting that genetic factors contribute to the risk of developing the condition (Burnier and Wuerzner, 2015). Multiple genetic variants have been identified, including those involved in the renin-angiotensin-aldosterone system (RAAS), sodium transport, and vascular tone regulation (Angeli et al., 2015). Lifestyle choices such as smoking, drinking, diet, stress, and sedentariness also cause damage to blood vessels which can elicit deleterious changes in peripheral resistance and blood pressure elevation (Bidani and Griffin, 2004).

**FIGURE 2:**
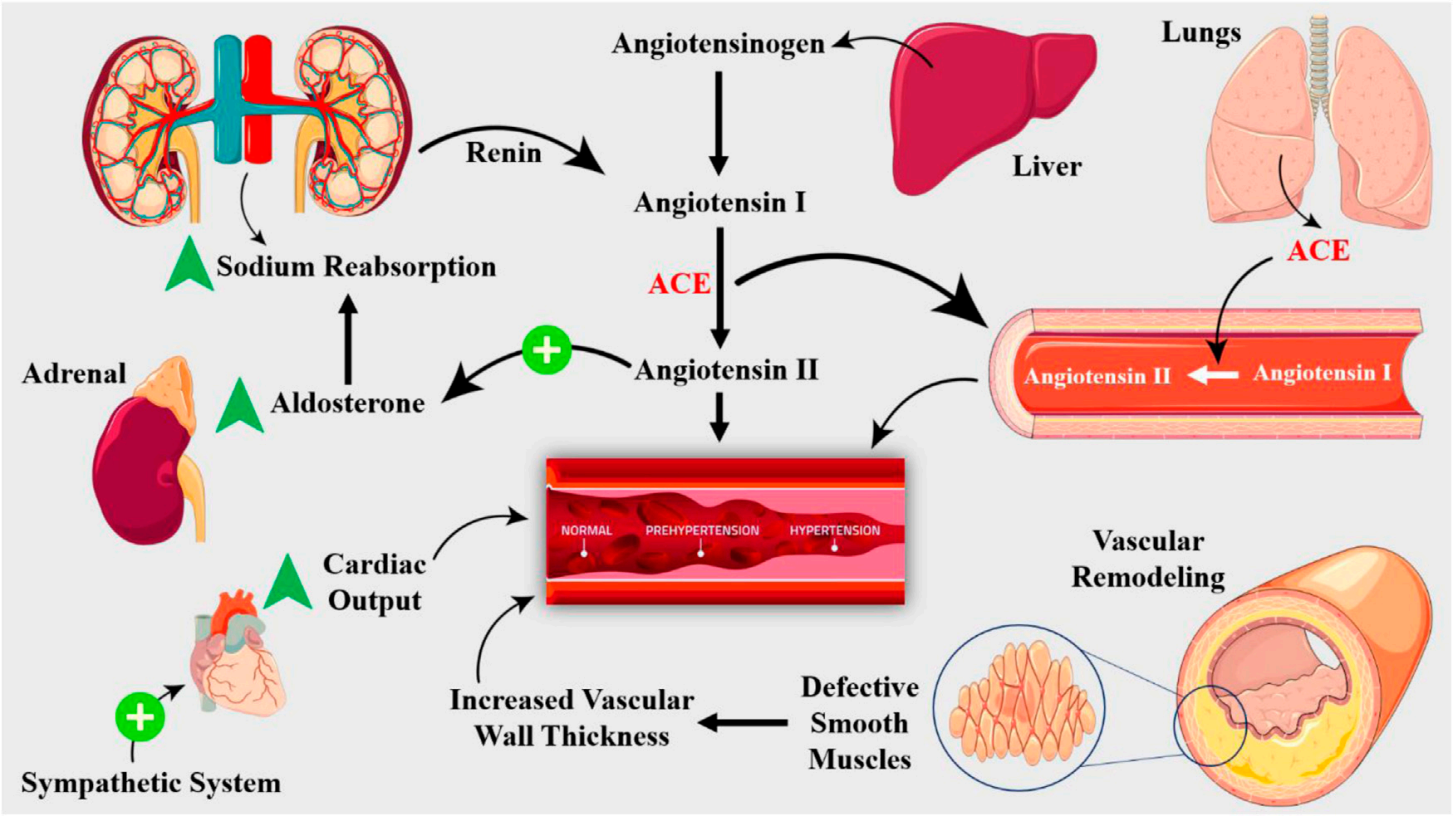
The RAAS is key player in hypertension pathogenesis. Renin, an enzyme produced by the kidneys, converts angiotensinogen to angiotensin I, which is then converted to angiotensin II by ACE, which is produced by the lungs. Angiotensin II is a potent vasoconstrictor that stimulates the release of aldosterone, a hormone that promotes sodium retention and potassium excretion. The RAAS is activated in response to decreased renal blood flow, decreased sodium delivery to the distal tubule, or increased sympathetic activity. Structural changes in the vasculature, including remodeling and stiffening, contribute to the development of hypertension. Vascular smooth muscle cell hypertrophy and hyperplasia lead to increased wall thickness, reducing the lumen diameter and increasing peripheral resistance.

The kidneys have a critical role in blood pressure regulation. Renal dysfunction, such as nephrosclerosis, glomerulonephritis, and renal artery stenosis, can lead to hypertension (Figure 2). The kidneys regulate blood pressure through the RAAS. This system causes vasoconstriction, sodium retention, and aldosterone production, leading to blood pressure elevation. The endothelium of blood vessels also plays a critical role in regulating vascular tone (Braunthal and Brateanu, 2019; Rodrigo et al., 2011). In hypertension, dysfunctional endothelium leads to impaired vasodilation, increased peripheral resistance, increased vasoconstriction, and blood pressure elevation. Endothelial dysfunction and consequent vascular remodeling is hallmark of hypertensive disorders (Hogg et al., 2022). Chronic hypertension leads to vascular remodeling, characterized by increased media thickness, decreased lumen diameter, and increased collagen deposition, leading to increased peripheral resistance and blood pressure elevation (Gibbins et al., 2016). Cardiac factors, such as left ventricular hypertrophy and diastolic dysfunction, can also contribute to the development of hypertensive disorders. Chronic hypertension leads to cardiac remodeling, characterized by increased left ventricular mass and decreased compliance, exacerbating the increased peripheral resistance and blood pressure elevation (Phoswa and Khaliq, 2021). Balancing vasoconstriction, sodium chloride retention, and aldosterone production, neurohumoral factors—such as the sympathetic nervous system and the renin-angiotensin-aldosterone system—are also essential for controlling blood pressure (Zanatta et al., 2019).

While the underlying causes of hypertension are still not fully understood, recent advances in molecular biology and physiology have shed light on the critical role of molecular signaling and homeostasis in the development and progression of hypertensive disorders (Viau et al., 2015). The RAAS is a key molecular signaling pathway activated in response to decreased blood pressure, leading to the release of renin from the juxtaglomerular cells of the kidney (Saxena et al., 2018). Angiotensinogen becomes transformed into angiotensin I by renin, and the ACE subsequently transforms this angiotensin I into angiotensin II (Figure 2). A strong vasoconstrictor, angiotensin II raises blood pressure by inducing vascular smooth muscle cells to contract. Furthermore, angiotensin II encourages the adrenal gland to generate aldosterone, which improves sodium reabsorption in the kidneys, raising blood pressure even further (Carey, 2008). A strong vasodilator, nitric oxide (NO) is essential for controlling blood pressure. Endothelial nitric oxide synthase (eNOS) produces NO from L-arginine in response to a number of stimuli, such as shear stress, acetylcholine, and Bradykinin (Townsend et al., 2016). Cyclic guanosine monophosphate (cGMP) is produced when NO diffuses to nearby vascular smooth muscle cells and activates soluble guanylyl cyclase. NO generation is frequently compromised in hypertensive diseases, which results in reduced vasodilation and elevated blood pressure (Harrison et al., 2021). In hypertensive diseases, epigenetic changes—such as DNA methylation and histone modification—are essential for controlling gene expression. Genes involved in blood pressure regulation, including the RAAS and NO signaling pathways, can have their expression altered by epigenetic changes (DeMarco et al., 2014). For example, DNA methylation of the promoter region of the ACE gene has been shown to contribute to increased ACE expression and activity in hypertensive individuals (Narang and Szymanski, 2021). In addition, sympathetic nervous system (SNS) modulates vascular resistance and cardiac output, while the baroreceptor reflex, a negative feedback mechanism, responds to changes in blood pressure by adjusting heart rate and vasomotor tone (Palei et al., 2013).

## 3 The role of ECM in stability and cellular communication and its pathophysiology in diseases

The ECM is composed of a diverse range of biomolecules, including collagens, proteoglycans, glycoproteins, and elastin, which are secreted by cells and assembled into a highly organized three-dimensional structure (Ryan, 2009). The ECM is essential for preserving tissue integrity, controlling cellular migration and differentiation, and adjusting how cells react to biochemical and mechanical stimuli (Valiente-Alandi et al., 2016; Karamanos et al., 2021). One of the primary functions of the ECM is to provide mechanical stability to tissues. The ECM acts as a scaffold, allowing cells to adhere, migrate, and proliferate. The ECM’s mechanical properties, such as its elasticity, stiffness, and viscoelasticity, influence cell behavior and fate (Schlie-Wolter et al., 2013; Walker et al., 2018). For instance, the ECM’s stiffness can regulate stem cell differentiation, with stiffer matrices (generally refers to the rigidity or stiffness of a structure) promoting osteogenic differentiation and softer matrices favoring adipogenic differentiation (Hansen et al., 2015). Furthermore, the ECM’s mechanical properties can influence cell migration, with cells migrating more efficiently on stiffer matrices. In addition to providing mechanical stability, the ECM also plays a critical role in cellular communication (Kudo and Kii, 2018). Growth factors, cytokines, and other signaling molecules are stored in the ECM and can be released in response to microenvironmental changes. These signaling molecules can bind to specific receptors on the surface of cells, triggering various cellular responses, such as proliferation, differentiation, and migration. The ECM can also modulate cellular behavior by presenting adhesive ligands, such as integrins, which can activate signaling pathways that regulate cellular function (Bowers et al., 2010; Marastoni et al., 2008; Pupa et al., 2002).

The ECM’s role in cellular communication is also highlighted by its ability to regulate the activity of various signaling pathways. The ECM can modulate the activity of growth factor receptors, such as the epidermal growth factor receptor (EGFR), by sequestering growth factors or presenting them to cells in a spatially and temporally regulated manner (Walma and Yamada, 2020; Clause and Barker, 2013). The ECM can also regulate the activity of integrin-mediated signaling pathways, which are critical for cell adhesion, migration, and survival. Dysregulation of the ECM’s role in stability and cellular communication can contribute to various diseases (Li J. et al., 2020). For instance, changes in the mechanical characteristics of the ECM might play a role in the development of fibrotic disorders such idiopathic pulmonary fibrosis, where the ECM becomes overly rigid and scar tissue is deposited. Similar to this, alterations in the form and composition of the ECM can aid in the development of cancer by encouraging the migration, invasion, and metastasis of tumor cells (Lynch and Matrisian, 2002; Watt and Huck, 2013).

The ECM degradation significantly in hypertension, which aids in the onset and progression of disease. These changes include increased deposition of collagen, fibronectin, and other matrix proteins, leading to vascular stiffening, remodeling, and inflammation (Ma et al., 2012). These changes impair endothelial function, increase vascular resistance, and promote the development of hypertension. One of the key mechanisms by which the ECM contributes to hypertension is through the regulation of vascular tone (Sainio and Järveläinen, 2020). The ECM provides a scaffold for vascular smooth muscle cells (VSMCs) and endothelial cells, and its composition and organization influence the contraction and relaxation of VSMCs, thereby modulating blood vessel diameter and blood pressure (Urbanczyk et al., 2020).

## 4 MMPs biochemistry, classification and physiological function

MMPs are synthesized as inactive zymogens, which undergo proteolytic activation to become catalytically active enzymes. The active site of MMPs contains a zinc ion, which is essential for their catalytic activity. The zinc ion is coordinated by three histidine residues, and the enzyme’s activity is regulated by the binding of tissue inhibitors of metalloproteinases (TIMPs). The typical structure of MMPs consists of an N-terminal zymogenic propeptide domain (∼80 amino acids), a metal-dependent catalytic domain (∼170 amino acids), a linker region (∼15–65 amino acids), and a C-terminal hemopexin-like domain (∼200 amino acids). The catalytic domain contains a conserved zinc-binding motif, essential for metalloproteinase activity, while the propeptide domain contains a cysteine switch motif that maintains the enzyme in an inactive state until cleavage occurs (Dzobo and Dandara, 2023; Vandooren et al., 2013; Cui et al., 2017; Cauwe and Opdenakker, 2010).

**FIGURE 3:**
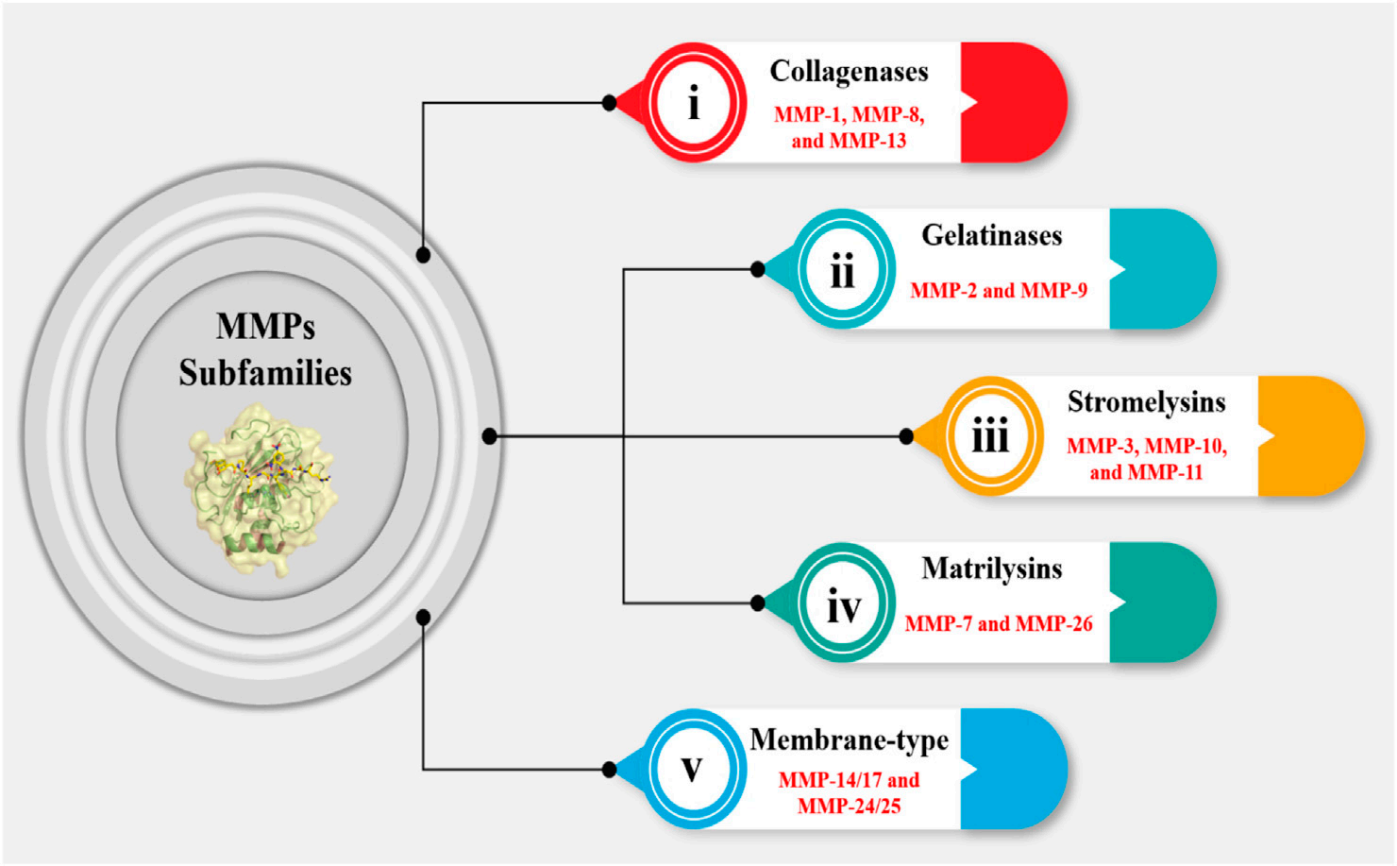
MMPs are responsible for degrading ECM components. The MMP subfamily consists of 24 members, which are categorized into five groups. The first group, comprising MMP-1, MMP-8, and MMP-13, is involved in collagen degradation (collagenase subgroup). The second group, consisting of MMP-2 and MMP-9, is characterized by their ability to degrade gelatin (gelatinase subgroup). The third group, which includes MMP-3, MMP-10, and MMP-11, is involved in the degradation of a wide range of ECM components (stromelysin subgroup). The fourth group, comprising MMP-7 and MMP-26, is primarily involved in the degradation of ECM components in the lung (matrilysin subgroup). The fifth group is essential in various physiological processes, including wound healing and embryonic development, as well as in pathological conditions such as cancer metastasis and cardiovascular diseases.

MMPs can be classified into several subfamilies based on their structure, function, and substrate specificity including (Figure 3): (i) Collagenases (MMP-1, MMP-8, and MMP-13): These enzymes are responsible for degrading collagen; (ii) Gelatinases (MMP-2 and MMP-9): These enzymes degrade denatured collagen and are involved in the remodeling of the ECM during tissue repair and cancer progression; (iii) Stromelysins (MMP-3, MMP-10, and MMP-11): These enzymes have a broader substrate specificity and are involved in the degradation of a variety of ECM components, including collagen, laminin, and fibronectin; (iv) Matrilysins (MMP-7 and MMP-26): Collagen, laminin, and elastin are among the ECM components that are broken down by these enzymes; (v) Membrane-type MMPs (MT-MMPs): Including MMP-14, MMP-15, MMP-16, MMP-17, MMP-24, and MMP-25, are anchored to the cell membrane and have distinct regulatory mechanisms involving furin cleavage sites in their pro-peptides. These MMPs are crucial for cell migration, invasion, and the activation of pro-MMPs, thus participating actively in various physiological and pathological processes (Vempati et al., 2007; Bellayr et al., 2009; Cauwe et al., 2007).

The physiological functions of MMPs are diverse and multifaceted, and their dysregulation has been implicated in various diseases. One of the primary physiological functions of MMPs is to facilitate cell migration and tissue remodeling. During embryonic development, MMPs are essential for the migration of cells and the formation of tissues (Gerlach et al., 2005). For instance, during neural tube development, MMP-2 and MMP-9 contribute to the migration of neural crest cells. MMPs are necessary for the remodeling of tissues in adults in response to inflammation or damage. For example, during wound healing, MMP-1 and MMP-3 contribute to the breakdown of ECM components, which permits keratinocytes and fibroblasts to migrate to the wound site (Palei et al., 2008). MMPs also play a critical role in angiogenesis and vascular remodeling. During angiogenesis, MMPs are involved in the degradation of the ECM. MMP-2, MMP-9, and MMP-14 are all involved in this process, and their dysregulation has been implicated in various vascular disorders (Ma et al., 2014). Additionally, MMPs, particularly MMP-2 and MMP-9, are involved in degradation of ECM components in the vascular wall (such as collagen, laminin and aggrecan), allowing adaptation of blood vessels to changes in flow and/or blood pressure (Souza-Tarla et al., 2005). A complicated interaction between transcriptional, post-transcriptional, and post-translational pathways controls expression and activity of MMPs. At the transcriptional level, MMPs are regulated by transcription factors such as activator protein-1 (AP-1), nuclear factor kappa B (NF-κB), and specificity protein-1 (SP-1). At the post-transcriptional level, MMPs are regulated by microRNAs, which can bind to the 3′untranslated region of MMP mRNAs, leading to their degradation (Zamilpa et al., 2012; Klein and Bischoff, 2011). At the post-translational level, MMPs are regulated by protein-protein interactions, such as the binding of TIMPs to MMPs, leading to their inhibition (Hijova, 2005).

MMPs are also involved in immune cell function, particularly in the migration and activation of immune cells. For instance, MMP-9 is involved in the migration of neutrophils to sites of inflammation, where they play a critical role in the elimination of pathogens (Śliwowska and Kopczyński, 2005). Additionally, MMP-2 and MMP-14 are involved in the activation of T-cells, which are essential for the adaptive immune response. During tissue repair, MMPs are involved in the degradation of ECM components, allowing for the migration and proliferation of cells to the site of injury (Karagiannis and Popel, 2004). However, excessive MMP activity can lead to the formation of scar tissue, which can impair tissue function (de Almeida et al., 2022). Additionally, MMP-2 and MMP-14 are involved in the degradation of ECM components in the brain, which is essential for the clearance of beta-amyloid plaques, a hallmark of Alzheimer’s disease. MMPs have been linked to a variety of cancer types, where they contribute to the growth and spread of tumors. (Jabłońska-Trypuć et al., 2016). MMP-2, MMP-9 and MMP-14 are also involved in the degradation of ECM components, permitting migration and invasion of cancer cells (Starr et al., 2012).

### 4.1 The role of MMPs on vascular function and structure

**FIGURE 4:**
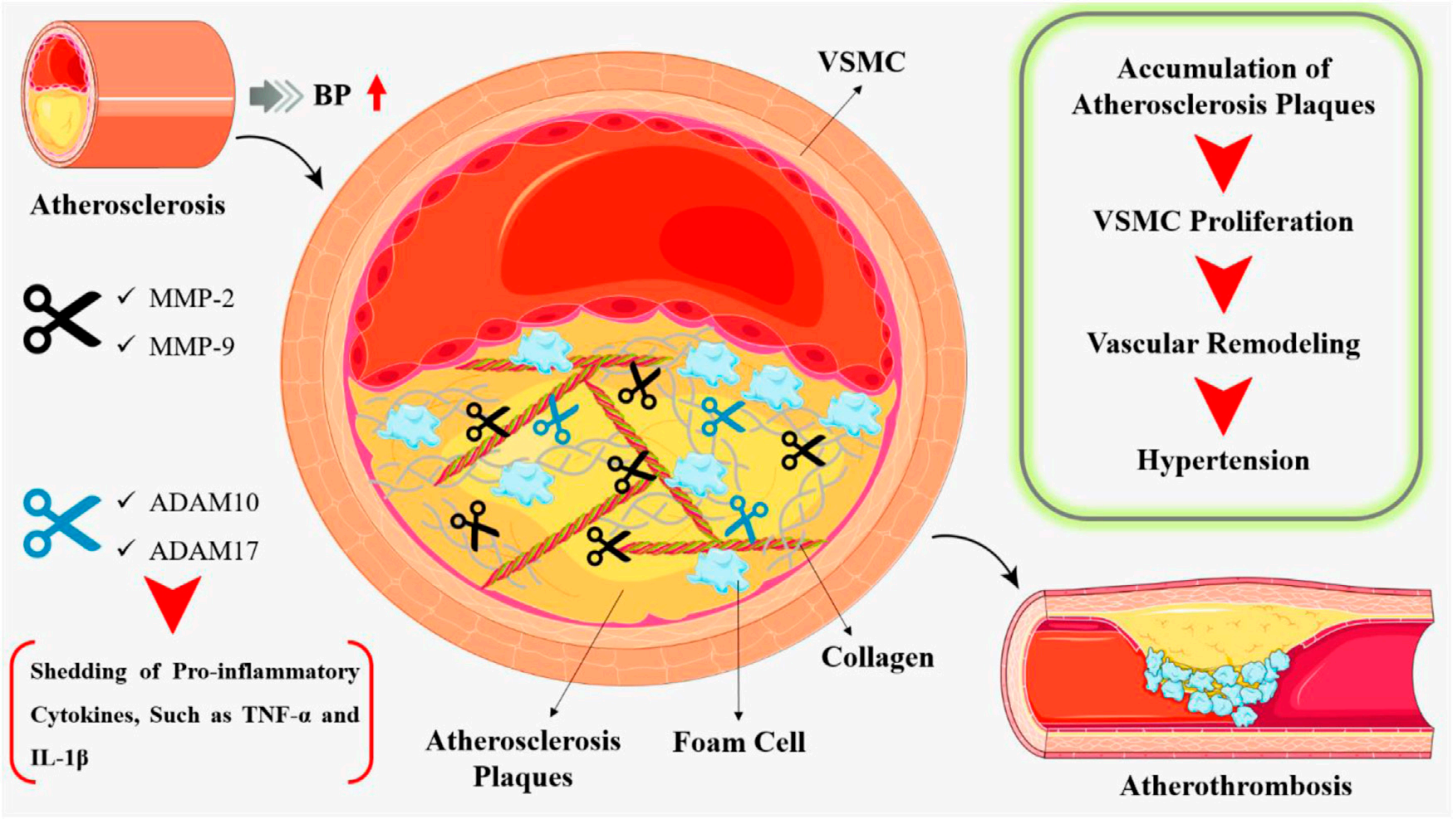
Atherosclerosis is a chronic inflammatory disease characterized by the accumulation of lipids, inflammatory cells, and fibrous tissue in the arterial wall, leading to the formation of atherosclerotic plaques. During the early stages of atherosclerosis, MMPs facilitate the migration of inflammatory cells into the arterial wall. MMPs, such as MMP-2 and MMP-9, degrade the extracellular matrix, allowing for the migration of these cells and the formation of atherosclerotic lesions. In addition, pro-inflammatory cytokines such as TNF-α and IL-1β, which exacerbate the inflammatory response in atherosclerosis, have been linked to the ADAM family, particularly ADAM10 and ADAM17.

In the context of cardiovascular disease, MMPs have been implicated in both the progression and regression of atherosclerosis, cardiac remodeling, and vascular inflammation. Atherosclerosis is defined by the accumulation of lipids, inflammatory cells, and fibrous tissue in the artery wall (Chen and Parks, 2009). MMPs have been implicated in various stages of atherosclerosis (Löffek et al., 2011), for example, MMP-2 and MMP-9 have been demonstrated to play a major role in the breakdown of the vascular ECM (increased degradation of elastin in the aortic wall), hence contributing to the instability of atherosclerotic plaques. (Siefert and Sarkar, 2012). The ADAM family, especially ADAM10 and ADAM17, has been implicated in the shedding of pro-inflammatory cytokines, such as tumour necrosis factor-alpha (TNF-α) and interleukin-1 beta (IL-1β) (Figure 4), which exacerbate the inflammatory response in atherosclerosis (Raffetto and Khalil, 2008).

Further, human aortic intima proteins including collagen, elastin, and proteoglycans play a vital role in regulation of vascular tone, blood pressure, and the transport of nutrients and oxygen to the tissues. In atherosclerosis, these proteins are subjected to degradation by MMPs, leading to the weakening of the atherosclerotic plaque (Wang and Khalil, 2018). The degradation of the aortic intima proteins also leads to the loss of vascular tone, resulting in increased blood pressure and cardiac workload. Furthermore, the degradation of the activating protein-1, also leads to the decreased transport of nutrients and oxygen to the tissues, resulting in tissue ischemia and necrosis (Kuzuya and Iguchi, 2003). MMPs also have been shown to play a crucial role in vascular inflammation by degrading the endothelial barrier, promoting the migration of inflammatory cells, and regulating the activity of pro-inflammatory cytokines. MMP-2, MMP-9, and ADAM17 have been implicated in the degradation of the endothelial barrier, leading to the increased permeability of the endothelium and the recruitment of inflammatory cells, which further promotes progression of vascular remodeling and hypertension (Merchant and Davidge, 2004; Prado et al., 2021). Vascular remodeling is characterized by VSMC proliferation, migration, and ECM deposition (Galis and Khatri, 2002). MMP-2 and MMP-9 have been shown to promote VSMC proliferation and migration, while membrane-type I matrix metalloproteinase (MT1-MMP) inhibits these processes (Hobeika et al., 2007). MMPs also regulate ECM deposition and degradation in hypertensive vessels. MMP-2 and MMP-9 can degrade collagen and elastin, leading to vascular stiffening and increased blood pressure. In contrast, MT1-MMP promotes the degradation of fibronectin, a pro-inflammatory ECM protein that contributes to vascular remodeling (Papazafiropoulou and Tentolouris, 2009).

One of the main routes by which MMPs exert their vascular actions is through the modulation of vasodilation. Vasodilation is a critical process that helps to maintain blood pressure, regulate blood flow, and ensure proper oxygenation of tissues. NO is a potent vasodilator that plays a central role in the regulation of blood vessel tone (Chen et al., 2013). MMPs can degrade the ECM, leading to the release of growth factors and cytokines that stimulate NO production (Chew et al., 2004). For example, MMP-2 and MMP-9 have been shown to cleave and activate PDGF, which is a potent stimulator of NO production (Hu et al., 2007). Additionally, MMPs can also cleave and activate the NO receptor, soluble guanylate cyclase, leading to an increase in NO production (Castro et al., 2011). On the other hand, NO can also regulate MMPs activity. NO has been shown to inhibit the activity of MMP-2 and MMP-9, likely through the S-nitrosylation of critical cysteine residues (Watanabe et al., 2018). S-nitrosylation is a post-translational alteration that involves the covalent attachment of a nitric oxide group to a cysteine residue, which inhibits enzyme function. NO can also induce the expression of TIMPs (Ketelhuth and Bäck, 2011). In the context of atherosclerosis, MMPs and NO play critical roles in the regulation of plaque stability and rupture. MMPs can degrade the fibrous cap, leading to plaque rupture and thrombosis, while NO can promote vasodilation and reduce blood pressure, thereby reducing the risk of plaque rupture (M Castro and E Tanus-Santos, 2013). MMPs and NO are involved in the remodeling of tissues during development, growth, and repair. Also, MMPs can activate pro-inflammatory cytokines, such as TNF-α, which can stimulate NO production, leading to vasodilation and increased blood flow to the site of inflammation (Johnson et al., 2011). MMPs also regulate Ang II signaling by degrading its receptor, angiotensin II type 1 receptor (AT1R) (Burbridge et al., 2002). Studies have shown that MMP-2 and MMP-9 are upregulated in response to Ang II, leading to increased degradation of AT1R and subsequent decreased blood pressure. However, this negative feedback loop is disrupted in hypertension, leading to increased Ang II signaling and vasoconstriction (Azevedo et al., 2014; Lenglet et al., 2013). In addition, MMPs regulate NO signaling by degrading the NO synthase enzyme, leading to decrease NO production. Studies have shown that MMP-2 and MMP-9 are upregulated in response to NO, leading to decreased NO production and subsequent increased blood pressure (Padmanabhan Iyer et al., 2016). Also, MMPs degrade various pro-inflammatory cytokines, including TNF-α and IL-1β, leading to decreased inflammation and subsequent decreased blood pressure. However, this anti-inflammatory effect is disrupted in hypertension, leading to increased inflammation and tissue damage (Ahmed et al., 2006).

## 5 MMPs and renal disorders

In renal fibrosis, MMPs have been implicated as key regulators of ECM turnover, cell-matrix interactions, and tissue remodeling. MMPs have been regularly shown to be elevated in several types of renal fibrosis, including unilateral ureteral obstruction (UUO), aristolochic acid nephropathy, and diabetic nephropathy (Jacob et al., 2001). In human renal biopsies, MMPs have been detected in fibrotic lesions, including glomeruli, tubules, and interstitium. The expression of MMPs is not limited to a specific cell type, as they have been found in various renal cells, including fibroblasts, tubular epithelial cells, and podocytes (Zakiyanov et al., 2019). The regulation of MMPs in renal fibrosis is complex and involves multiple signaling pathways. The transforming growth factor-beta (TGF-β) signaling pathway is a key regulator of MMP expression in renal fibrosis (Lenz et al., 2000). TGF-β stimulates the expression of MMP-2 and MMP-9, which are critical for ECM degradation and fibrosis progression. Additionally, the mitogen-activated protein kinases (MAPK) and phosphatidylinositol 3-kinase/protein kinase B (PI3K/Akt) signaling pathways have been implicated in the regulation of MMP expression in renal fibrosis (Catania et al., 2007).

MMPs also regulate the activity of various growth factors and cytokines, including TGF-β, platelet-derived growth factor (PDGF), and connective tissue growth factor (CTGF), which are critical for fibrosis progression (Narula et al., 2018). Furthermore, MMPs have been implicated in the regulation of epithelial-mesenchymal transition (EMT), a process in which epithelial cells acquire a mesenchymal phenotype, contributing to fibroblast accumulation and ECM production. MMPs have also been shown to regulate the activity of immune cells, including macrophages and T cells, which play a critical role in the development of renal fibrosis (Zhao et al., 2013).

The well-studied MMPs in the context of nephropathy are MMP-2, MMP-9, and MT1-MMP. MMP-2 and MMP-9 are gelatinases that degrade collagen IV, a key component of the glomerular basement membrane (GBM) (Zakiyanov et al., 2021). MT1-MMP is a membrane-type MMP that activates MMP-2 and degrades a range of ECM proteins. MMPs can also activate or inactivate other proteases, growth factors, and cytokines, highlighting their complex role in regulating the ECM and cellular behavior (Garcia-Fernandez et al., 2020). In nephropathy, MMPs are often overexpressed or aberrantly activated, leading to excessive ECM degradation and disruption of the glomerular filtration barrier. The expression of MMPs is regulated by a range of signaling pathways, including the MAPK and PI3K pathways. In addition, MMPs can be induced by pro-inflammatory cytokines, such as TNF-α and IL-1β, which are commonly elevated in nephropathic states (Sampieri and Orozco-Ortega, 2018).

In glomerulonephritis, MMPs contribute to the degradation of the GBM, leading to proteinuria and glomerular scarring. In diabetic nephropathy, MMPs are activated in response to hyperglycemia and oxidative stress, promoting the degradation of ECM proteins and the development of fibrosis (Tan and Liu, 2012). MMPs also involved in the development of glomerular hypertrophy, a common feature of chronic kidney disease (CKD). In this situation, MMPs contribute to the degradation of the ECM, leading to the expansion of the mesangial matrix and the development of glomerular sclerosis (Provenzano et al., 2020). In acute kidney injury (AKI), MMPs contribute to the degradation of the tubular basement membrane, leading to the loss of tubular integrity and the development of fibrosis. Pro-MMP-2 is converted into its active form by MMP-24, which is increased in the tubular epithelium in human diabetic kidney disease (DKD). Proximal, distal, and collecting tubules that were positive for MT5-MMP (MMP-24) usually showed tubular atrophy, likely resulting from DN progression (Friese et al., 2009; Liu et al., 2020). Also in CKD, MMPs promote the degradation of the ECM, leading to the development of interstitial fibrosis and the loss of renal function. An imbalance in the MMP/TIMP ratio, often due to decreased MMPs or increased TIMPs, results in ECM accumulation, promoting CKD progression, and increased MMP-9 activity in CKD, even in its early stages. This activity leads to structural alterations in the renal tubule and glomerulus, particularly in advanced stages of CKD when patients develop severe renal fibrosis (AlQudah et al., 2020).

## 6 MMPs and RAAS

**FIGURE 5:**
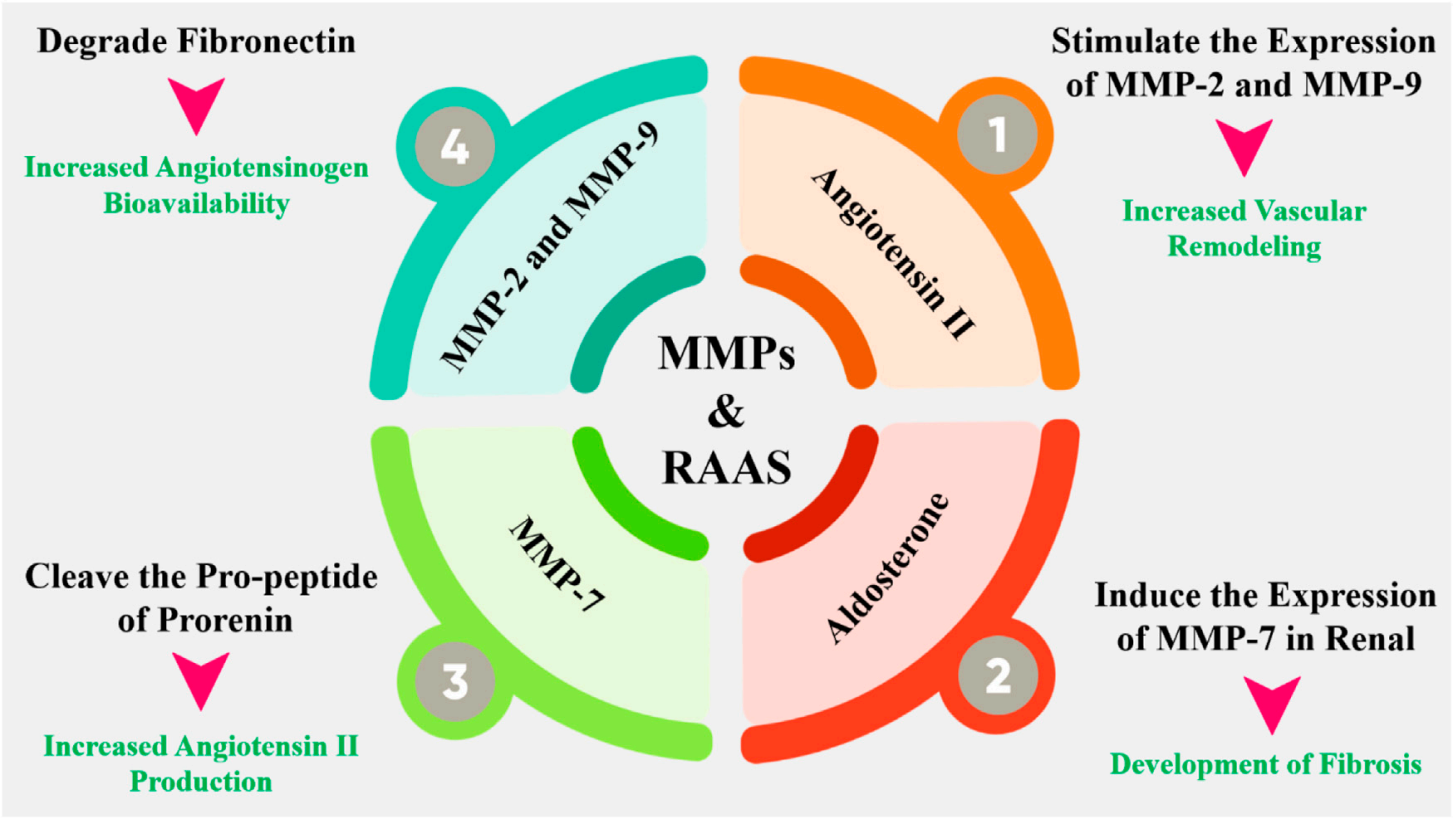
The interplay between MMPs and RAAS is complex and bidirectional. MMPs can regulate the RAAS by degrading or activating key components of the system, such as angiotensinogen and ACE. Conversely, the RAAS can modulate MMP activity by regulating the expression and activity of MMPs. Additionally, MMPs have been shown to regulate the expression of RAAS components, such as the angiotensin II type 1 receptor (AT1R). Angiotensin II has been shown to stimulate the expression and activity of MMPs, such as MMP-2 and MMP-9, in various cell types, including vascular smooth muscle cells and fibroblasts. This can lead to excessive ECM degradation and remodeling, contributing to the development of cardiovascular disease.

The interplay between MMPs and RAAS is multifaceted and bidirectional. MMPs can influence RAAS activity by degrading ECM components that regulate the bioavailability of RAAS components (Duprez, 2006). For example, MMP-2 and MMP-9 have been shown to degrade fibronectin, a protein that binds to angiotensinogen, thereby increasing its bioavailability (Figure 5). Additionally, MMP-7 can cleave the propeptide of prorenin, activating the enzyme and increasing angiotensin II production (Muñoz-Durango et al., 2016). Also, RAAS components can modulate MMP activity and expression. Angiotensin II, for instance, can stimulate the expression of MMP-2 and MMP-9 in vascular smooth muscle cells, leading to increased ECM degradation and vascular remodeling (Ishibashi et al., 2010). Aldosterone, another key component of RAAS, can induce the expression of MMP-7 in the kidney, contributing to the development of fibrosis. In hypertensive situations, the activation of RAAS leads to increased MMP-2 and MMP-9 expression, which in turn contributes to vascular remodeling and stiffening (Su et al., 2019). Similarly, in cardiac fibrosis, the activation of MMPs by RAAS components leads to ECM degradation and fibrosis, exacerbating cardiac dysfunction. Targeting the MMP-RAAS axis has emerged as a possible treatment approach for a variety of disorders. Doxycycline and other MMP inhibitors have been found to minimize cardiac fibrosis and enhance heart function in animal models of hypertension (Wu et al., 2020). Moreover, the MMP-RAAS axis has been implicated in various other diseases, including atherosclerosis, chronic kidney disease, and liver fibrosis. In atherosclerosis, for example, the activation of MMPs by RAAS components contributes to the degradation of ECM and the formation of vulnerable plaques (Mora-Gutiérrez et al., 2020). In chronic kidney disease, RAAS activation induces MMP expression, which contributes to the development of fibrosis and renal failure. The MMP-RAAS axis serves a vital function in controlling tissue morphogenesis and patterning (Lods et al., 2003). During embryonic development, the coordinated activity of MMPs and RAAS components is essential for the formation of various tissues and organs. Dysregulation of the MMP-RAAS axis during development has been implicated in various congenital disorders, including cardiac defects and craniofacial abnormalities (Rabkin, 2017).

## 7 MMPs and natriuretic system and catecholamine

**FIGURE 6:**
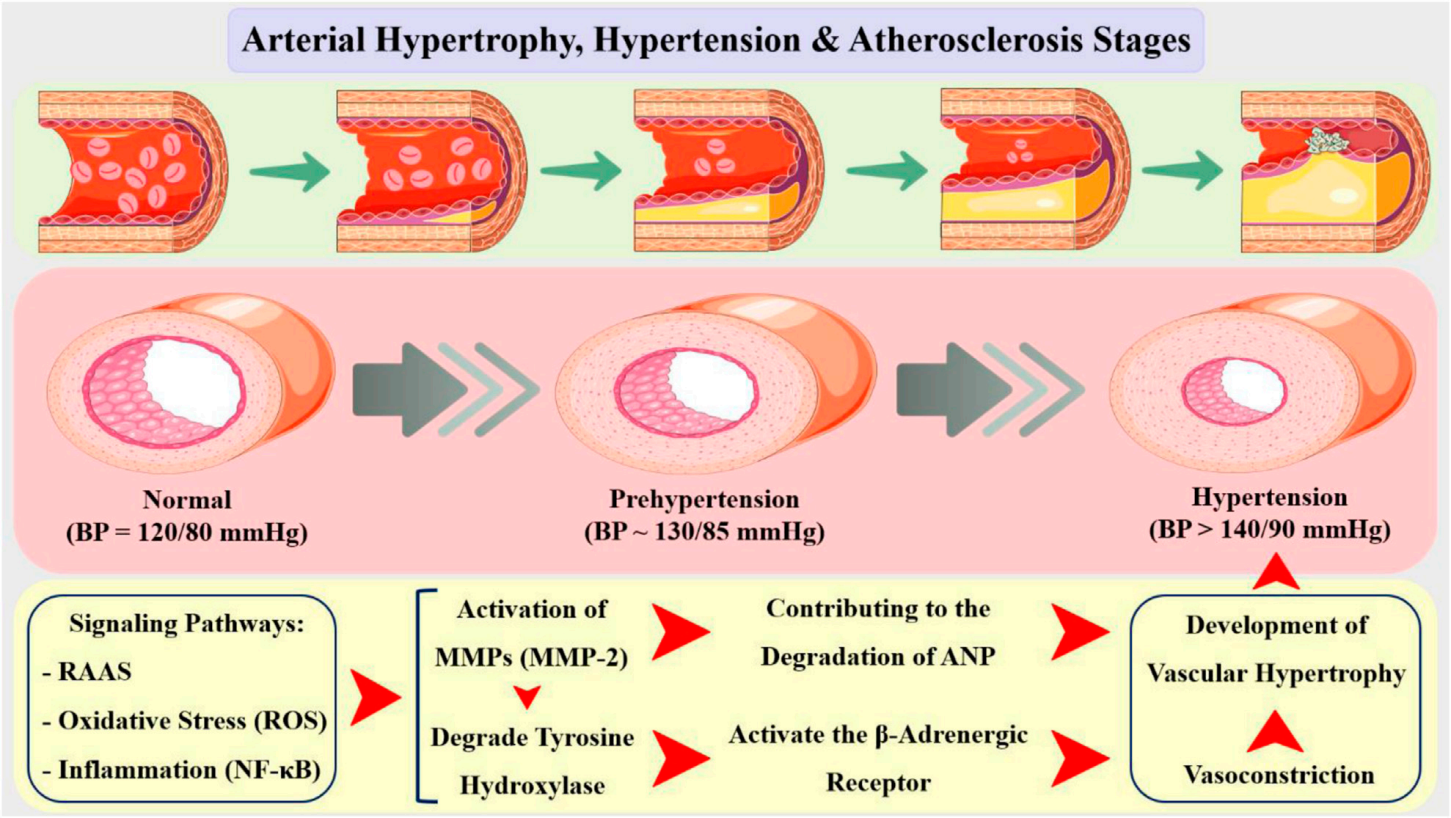
Arterial hypertrophy is a common feature of various cardiovascular diseases, including hypertension, atherosclerosis, and restenosis. The relationship between arterial hypertrophy and hypertension is bidirectional, with each condition exacerbating the other. On one hand, hypertension can induce arterial hypertrophy by stimulating the proliferation of VSMCs and promoting the production of growth factors. On the other hand, arterial hypertrophy can contribute to the development of hypertension by increasing peripheral resistance and reducing vascular compliance. In addition, the increased blood pressure can cause endothelial dysfunction, which leads to the activation of inflammatory pathways and the recruitment of immune cells, ultimately resulting in the formation of atherosclerotic plaques. Additionally, hypertension can also contribute to the rupture of these plaques, leading to acute cardiovascular events. In the context of arterial hypertrophy, the activity of MMPs is regulated by various signaling pathways, including the RAAS, oxidative stress, and NF-κB pathway. These signaling pathways regulate the expression and activity of MMPs, leading to the degradation of ECM components and the development of arterial hypertrophy. MMP-2 have been implicated in the development of arterial hypertrophy and expressed by VSMCs and is involved in the degradation of collagen and elastin, leading to the loss of arterial wall elasticity and compliance.

The atria produce atrial natriuretic peptide (ANP), a powerful vasodilator and natriuretic hormone that plays an important role in blood pressure management and cardiovascular homeostasis. In hypertension, ANP levels are frequently reduced contributing to the development and progression of cardiovascular disease (Belo et al., 2016). Recent studies have demonstrated that MMPs, particularly MMP-2 and MMP-9, are upregulated in hypertensive vessels, contributing to the degradation of ANP and the development of vascular hypertrophy (Figure 6). MMP activation has been demonstrated to be regulated by multiple signaling pathways, including the RAAS, oxidative stress, and inflammation, which are all known to be activated in hypertension (Gresele et al., 2017; Wallace et al., 2005).

Furthermore, MMP inhibition enhanced endothelial function and decreased vascular stiffness, suggesting that MMPs play a role in the development of endothelial dysfunction and vascular remodeling in hypertension (Chow et al., 2007). The results obtained imply that MMP inhibition could be a potential therapeutic approach to the treatment of cardiovascular disease, particularly in individuals with hypertension (Wang et al., 2015; Tsai et al., 2019; Wang et al., 2003). Results showed that MMP-2 and MMP-9 degraded ANP in a dose-dependent manner, and that MMP inhibition prevented ANP degradation and increased its biological activity (Parthasarathy et al., 2013). Moreover, MMPs regulated ANP expression at the transcriptional level, with MMP-2 and MMP-9 suppressing ANP gene expression in atrial myocytes (Okumura et al., 2011). These findings suggest that MMPs play a critical role in the regulation of ANP expression and activity, and that MMP inhibition may be a useful strategy for increasing ANP levels and activity in hypertension (Kalaiarasu et al., 2016; Kasama et al., 2008).

Catecholamines, such as norepinephrine, epinephrine, and dopamine, are neurotransmitters that play a vital role in the sympathetic nervous system’s response to stress and blood pressure regulation (Kawakami et al., 2004). In vascular hypertension, the equilibrium between catecholamine production and breakdown is broken, resulting in a rise in catecholamine levels, which exacerbates hypertension (Elmas et al., 2011; Speidl et al., 2004). Studies have shown that MMPs, particularly MMP-2 and MMP-9, are upregulated in hypertensive vessels (expression in VSMCs and endothelial cells), leading to the degradation of ECM components, including collagen and elastin. This degradation can result in the release of bound catecholamines, increasing their bioavailability and contributing to the development of hypertension (Chakroborty et al., 2009; Banfi et al., 2005; Rassler et al., 2012). Furthermore, MMPs can also regulate catecholamine levels by degrading the enzymes involved in catecholamine synthesis and degradation. For instance, MMP-2 has been shown to degrade tyrosine hydroxylase, a rate-limiting enzyme in catecholamine synthesis, leading to a decrease in catecholamine production (Shi et al., 2010; Yang et al., 2006). Conversely, MMP-9 has been found to degrade monoamine oxidase (MAO), an enzyme responsible for catecholamine degradation, resulting in increased catecholamine levels. In addition to their direct effects on catecholamine levels, MMPs can also influence catecholamine signaling pathways (Essa et al., 2012). MMP-2 has been shown to activate the β-adrenergic receptor, leading to an increase in catecholamine-mediated vasoconstriction and hypertension (Yang et al., 2008). Conversely, MMP-9 has been found to inhibit the α-adrenergic receptor, leading to a decrease in catecholamine-mediated vasoconstriction and a subsequent decrease in blood pressure (Yang et al., 2012; Zhang et al., 2017).

## 8 MMPs knockout models in hypertensive disorders

Research involving MMP knockout models in hypertensive contexts has provided valuable insights into the involvement of MMPs in vascular remodeling and organ damage associated with hypertension. A study investigated the function of MMP2 in the progression of proteinuria and renal damage following the induction of hypertension or diabetes in MMP2 knockout rats. The findings indicated that MMP2 knockout rats exhibited reduced levels of proteinuria, glomerular injury, renal fibrosis, and podocyte loss. Consequently, the absence of MMP-2 in Dahl salt-sensitive rats was associated with lower mean arterial pressure and diminished renal injury when compared to wild-type counterparts (Pushpakumar et al., 2013). A related investigation indicates that the deletion of MMP9 in hypertensive rat models reinstates the autoregulation of renal blood flow and mitigates the progression of hypertension, proteinuria, glomerular damage, and renal interstitial fibrosis. Conversely, MMP-9 knockout mice subjected to angiotensin II treatment demonstrated more pronounced elevations in blood pressure and diminished compliance of the carotid artery when compared to their wild-type counterparts. This suggests that MMP-9 may play a protective role in the early stages of hypertension by preserving vascular compliance (Yang et al., 2012).

The interactions between MMPs and other signaling pathways in the context of hypertension have been elucidated through knockout models. For example, MMP-9 has been shown to interact with the angiotensin II signaling pathway, where its activation contributes to the hypertensive response. In MMP-9 knockout mice, there is a notable reduction in angiotensin II-induced vascular remodeling, indicating that MMP-9 may facilitate the effects of this potent vasoconstrictor (Barhoumi et al., 2017). Furthermore, studies have demonstrated that MMPs can modulate the activity of other proteases and inflammatory mediators, creating a complex network of interactions that exacerbate hypertensive conditions. These findings suggest that targeting MMPs may not only directly influence vascular remodeling but also disrupt harmful interactions within the broader hypertensive signaling landscape, offering a multifaceted approach to hypertension management (Chakroborty et al., 2020; Li K. et al., 2020).

## 9 MMPs inhibitor as therapeutic agents for hypertension

Several MMP inhibitors have been developed and tested in preclinical models of hypertension. These inhibitors can be classified into several categories, including synthetic peptides, non-peptidic molecules, and natural products (Luchian et al., 2022). Synthetic peptides, such as batimastat and marimastat, have been shown to be effective in reducing blood pressure and improving vascular function in hypertensive animals (Cabral-Pacheco et al., 2020; Mondal et al., 2020). Non-peptidic molecules, such as doxycycline and minocycline, have also been shown to be effective in reducing blood pressure and improving vascular function in hypertensive animals (Napoli et al., 2020). Natural products, such as green tea extract and curcumin, have also been shown to have anti-hypertensive effects in preclinical models (Das et al., 2020). For example, green tea extracts reduced blood pressure and improved vascular function in spontaneously hypertensive rats (Zakiyanov et al., 2021). Another study found that curcumin reduced blood pressure and improved renal function in hypertensive rats (Das et al., 2021).

Recent studies suggest that antihypertensive therapies may influence MMP activity and thereby modify ECM metabolism and VSMC function (Chuliá-Peris et al., 2022; Broekaart et al., 2021). For example, patients treated with lisinopril or candesartan exhibited significant reductions in both BP and MMP-9 concentrations after 3 months of treatment, suggesting a potential for MMP inhibitors to serve dual purposes in managing hypertension and reducing organ damage (Chuliá-Peris et al., 2022; Broekaart et al., 2021; Laronha et al., 2020). The correlation between MMP activity and BP levels reinforces the therapeutic relevance of MMP modulation in hypertensive patients (Zipfel et al., 2020; Luddi et al., 2020). Furthermore, marimastat, a broad-spectrum MMP inhibitor, functions by mimicking the structure of natural MMP substrates, thereby binding to MMPs and preventing the degradation of the basement membrane. Marimastat has been shown to inhibit MMPs such as MMP-9, MMP-1, MMP-1, MMP-2, MMP-14, and MMP-7. Its inhibitory action prevents endothelial cell migration, which is necessary for new blood vessel formation, and also blocks tumor cells from entering or exiting blood vessels, thereby preventing metastasis (Gimeno et al., 2020; Kiuru et al., 2021; Todd et al., 2020; Sinno et al., 2013).

### 9.1 The modulatory effects of natural products on MMPs

Natural products, such as flavonoids, polyphenols, and omega-3 fatty acids, have been identified as potential MMP inhibitors. These compounds have been shown to inhibit MMP activity, reduce inflammation, and improve vascular function in both *in vitro* and *in vivo* studies (de Meijer et al., 2010; Goffin et al., 2005). The advantage of natural MMP inhibitors lies in their safety profile, as they are generally well-tolerated and have minimal off-target effects. Curcumin, a polyphenol extracted from turmeric, has been extensively studied for its antihypertensive properties. Curcumin has been shown to inhibit MMP-2 and MMP-9 activity, reduce inflammation, and improve endothelial function in animal models of hypertension (Islam et al., 2024; Krebber et al., 2020). Additionally, clinical studies have shown that curcumin is effective in decreasing blood pressure in hypertensive patients (Ronsisvalle et al., 2020; Saragusti et al., 2010). Resveratrol, a polyphenol found in grapes and berries, has been shown to inhibit MMP-2 and MMP-9 activity, reduce oxidative stress, and improve vascular function in animal models of hypertension (Lim and Kim, 2007). Omega-3 fatty acids, particularly eicosapentaenoic acid (EPA) and docosahexaenoic acid (DHA), have anti-inflammatory properties and have been shown to inhibit MMP-2 and MMP-9 activity (Crascì et al., 2018).

Quercetin, a flavonoid present in apples and onions, has been demonstrated to inhibit MMP-2 and MMP-9 activity in human umbilical vein endothelial cells. Similarly, epigallocatechin gallate (EGCG), a flavonoid contained in green tea, has been observed to suppress MMP-2 and MMP-9 production in human aortic smooth muscle cells (Cayetano-Salazar et al., 2022). These findings suggest that flavonoids may be useful in preventing or treating hypertension-related vascular remodeling and fibrosis (Ende and Gebhardt, 2004). The mechanisms by which flavonoids modulate MMP activity are not fully understood, but several pathways have been proposed. One possible mechanism involves the inhibition of MMP gene expression through the suppression of NF-κB and AP-1 transcriptional activity (Chojnacka and Lewandowska, 2018). Flavonoids may also inhibit MMP activity by binding to the active site of the enzyme, thereby blocking substrate access. Additionally, flavonoids may modulate MMP activity by altering the redox state of the cell, as MMPs are sensitive to oxidative stress (Annabi et al., 2002).

Animal studies have provided further evidence for the antihypertensive effects of flavonoids. For example, a study in spontaneously hypertensive rats showed that quercetin supplementation reduced blood pressure and improved cardiovascular function (Calabriso et al., 2016; Saavedra et al., 2016). Similarly, a study in mice with angiotensin II-induced hypertension found that EGCG supplementation reduced blood pressure and inhibited vascular remodeling (Hilliard et al., 2020). While the existing evidence suggests that flavonoids may be useful in preventing or treating hypertension, several limitations and challenges remain. One major challenge is the variability in flavonoid bioavailability and bioactivity, which can affect their efficacy in different individuals (Sim et al., 2007). Additionally, the optimal dosage and duration of flavonoid supplementation required to achieve therapeutic effects in hypertension are not well established. Flavonoids are a desirable therapeutic target for the prevention and treatment of hypertension because of their antihypertensive properties (Santos et al., 2015). Several potential therapeutic applications of flavonoids in hypertension can be envisioned by dietary supplementation pharmaceutical development and nutrigenomic approaches (Lee et al., 2018; Devipriya et al., 2007; Huang and Liaw, 2017).

### 9.2 α2-Macroglobulin

α2-Macroglobulin (alpha2M), as a plasma protein, is a significant protease inhibitor known for its ability to inhibit activated MMPs, particularly MMP-9. This glycoprotein, with a molecular weight of approximately 720 kDa, is primarily synthesized in the liver and plays a crucial role in regulating proteolytic activity in the human body (Agić et al., 2018; Matchett et al., 2006). Elevated levels of MMPs are often associated with pathological conditions, including cancer and inflammatory diseases, highlighting the importance of alpha2M in maintaining homeostasis. The interaction between alpha2M and MMPs has significant implications for diseases characterized by excessive proteolytic activity (Strek et al., 2007). For instance, in conditions like severe sepsis or cancer, where MMP-9 levels are elevated, α2M can help mitigate tissue damage by inhibiting these active proteases (Crascì et al., 2017). Additionally, understanding the dynamics of alpha2M’s interaction with MMPs could provide insights into therapeutic strategies targeting these pathways in various diseases (Wang et al., 2014; Tortorella et al., 2004).

The mechanisms underlying the antihypertensive effects of alpha2M are multifaceted and involve the inhibition of MMPs, reduction of vascular inflammation, and modulation of the renin-angiotensin-aldosterone system (Barcelona et al., 2013; Ikari et al., 2003). The inhibition of MMPs by alpha2M is a critical aspect of its antihypertensive effects (Cáceres et al., 2010). By binding to and inhibiting MMPs, alpha2M prevents the degradation of matrix proteins, thereby reducing vascular stiffness and improving blood flow. Additionally, alpha2M has been shown to inhibit the activity of other proteases, such as cathepsins and elastase, which are also involved in vascular remodeling and hypertension (de Laat-Kremers et al., 2024). Beyond its effects on proteolytic pathways, alpha2M has anti-inflammatory properties, which contribute to its antihypertensive effects. Vascular inflammation is a hallmark of hypertension, and alpha2M has been shown to reduce inflammation by inhibiting the activation of immune cells and the production of pro-inflammatory cytokines (Annapoorani et al., 2006). Additionally, by suppressing the ACE and decreasing the synthesis of angiotensin II, alpha2M has been shown to alter the renin-angiotensin-aldosterone pathway, a crucial blood pressure regulator (Rajamanickam et al., 2001). Studies showing that alpha2M is effective in lowering blood pressure and improving cardiovascular outcomes in animal models of hypertension further suggest its therapeutic promise in hypertension (Knopp, 2022; Oh et al., 2005). Additionally, alpha2M has been shown to have synergistic effects when combined with other antihypertensive agents, suggesting its potential as an adjunctive therapy in the management of hypertension (Raymond et al., 2009; Lagrange et al., 2022).

## 10 Techniques, advanced markers, and genetical modulators related to evaluation of MMPs function

Post-translational modifications (PTMs) of MMPs such as phosphorylation, ubiquitination, and glycosylation, have emerged as a critical regulatory mechanism that can alter the enzymatic activity, substrate specificity, and expression levels, thereby influencing their role in hypertension (Martos et al., 2018; Motomiya et al., 2003). Phosphorylation of MMP-2 has been shown to increase its activity, leading to enhanced degradation of type IV collagen and increased vascular permeability (Madzharova et al., 2019; Sun et al., 2024). Ubiquitination of MMP-2 has been shown to reduce its expression and activity, leading to decreased vascular remodeling and fibrosis (Leeming et al., 2011). Conversely, deubiquitination of MMP-9 and N-glycosylation of MMP-2 has been linked to increased expression and activity, contributing to vascular inflammation and remodeling (Liddy et al., 2013). In addition, O-glycosylation of MMP-9 has been linked to decreased activity and expression, reducing vascular inflammation and remodeling (Sawicki and Jugdutt, 2007; Ergul et al., 2004; Buelna-Chontal et al., 2021).

Epigenetic modifications, such as DNA methylation, histone modifications, and non-coding RNA regulation, significantly influence MMP expression and function in the context of hypertension (de Mello et al., 2019; Gajjala et al., 2015; Sarker et al., 2021; He et al., 2020). Studies have shown that histone modifications can alter the expression of MMPs in response to inflammatory cytokines, such as IL-1β, suggesting interplay between these modifications and MMP activity (Guarner-Lans et al., 2020; Cruz et al., 2021). Conversely, repressive histone marks can inhibit MMP expression, contributing to the pathophysiology of hypertension (Schiattarella et al., 2018), thus targeting histone-modifying enzymes could be a potential strategy for modulating MMP function (Weber et al., 2016). miRNAs can modulate the expression of MMPs directly by binding to their mRNA and inhibiting translation (Lacolley et al., 2020). For example, miR-146a has been shown to repress MMP-13 expression in chondrocytes, while miR-203 can enhance MMP-1 secretion in rheumatoid arthritis synovial fibroblasts. Similarly, lncRNAs can influence MMP expression by interacting with chromatin-modifying complexes and transcription factors, thereby regulating inflammatory responses (Chistiakov et al., 2017; Priviero, 2023).

Recent advancements in analytical techniques, including zymography, active-site probes followed by enzymatic digestion, and liquid chromatography-mass spectrometry (LC-MS) analysis have significantly enhanced the detection and quantification of MMPs in biological samples (Kupai et al., 2010). Zymography is effective for visualizing active enzymes, it may lack sensitivity compared to other methods (Hu and Beeton, 2010). LC-MS offers high sensitivity and specificity, it requires sophisticated equipment and expertise, making it less accessible for routine evaluations (Kotnik et al., 2018). Active-site probes followed by enzymatic digestion technique involves the application of small molecules that specifically bind to the active sites of MMPs, facilitating the study of their functional roles. However, standardization of this method remains a challenge, which can lead to variability in results (Xu et al., 2015).

Proteomic profiling, particularly through the use of the SOMAscan v3.1 platform, have significantly enhanced the evaluation of MMPs and their functions in hypertension. This innovative technology enables the simultaneous measurement of over 1,000 proteins, allowing for a comprehensive analysis of the proteomic landscape associated with cardiovascular health. The resulting data are processed to yield quantifiable measurements for a wide array of proteins, with annotations provided via established databases such as UniProt and Entrez Gene (Habet et al., 2023; Wort, 2021).

CRISPR/Cas9 gene-editing technology has emerged as a powerful tool for evaluating the function of MMPs in various biological contexts, including hypertension. This method allows for precise modifications of MMP genes, enabling researchers to investigate the specific roles these proteins play in disease mechanisms (Seidl et al., 2019). By designing guide RNAs (gRNAs) that target specific MMP genes, researchers can induce double-strand breaks that lead to gene disruption. This approach has proven effective in elucidating the functional contributions of MMPs in hypertension-related processes. The targeted editing allows scientists to assess changes in phenotype and gene expression, providing insights into the pathological roles of MMPs in hypertensive conditions (Camargo et al., 2023). For example, knock out of MMP-2 and MMP-9 by CRISPR/Cas9 system in animal models results in reduced hypertensive responses, highlighting its critical role in the development of hypertension (Waghulde, 2016; Wang et al., 2018).

## 11 Conclusion

Hypertension is a major public health concern and the pathophysiology of hypertension is complex, involving multiple cellular and molecular mechanisms. Among these, MMPs have emerged as key players in the regulation of blood pressure and vascular function. MMPs have been linked to the control of inflammation, remodeling, and vascular tone in the setting of hypertension. It has been demonstrated that MMP-2 and MMP-9 specifically contribute to the development of hypertension by cleaving extracellular matrix proteins, which results in vascular stiffness and elevated blood pressure. In hypertension, MMP-2 is upregulated, leading to increased ECM degradation and vascular remodeling. MMP-9, on the other hand, is primarily expressed in macrophages and has been implicated in the regulation of inflammation and immune responses. In hypertension, MMP-9 is also upregulated, contributing to the development of vascular inflammation and oxidative stress. Several pharmacological agents have been developed to target MMPs in hypertension. MMP inhibitors, such marimastat and doxycycline, are among these medications; in animal models of hypertension, they have been demonstrated to lower blood pressure and enhance vascular function. Furthermore, it has been demonstrated that a number of natural compounds, such as flavonoids and polyphenols, lower blood pressure and suppress MMP activity. The development of novel therapeutic strategies, including MMP inhibition, antioxidant therapy, exercise training, dietary modification, and gene therapy, may provide a promising approach for the treatment of hypertension.

## Glossary

MMPs: Metalloproteinases
ECM: Extracellular matrix
TIMPs: Tissue inhibitors of metalloproteinases
VSMCs: Vascular smooth muscle cells
BP: Blood pressure
NSAIDs: Nonsteroidal anti-inflammatory drugs
RAAS: Renin-angiotensin-aldosterone system
ACE: Angiotensin-converting enzyme
NO: Nitric oxide
eNOS: Endothelial nitric oxide synthase
cGMP: Cyclic guanosine monophosphate
SNS: Sympathetic nervous system
AP-1: Activator protein-1
SP-1: Specificity protein-1
UUO: Unilateral ureteral obstruction
PI3K/Akt: Phosphatidylinositol 3-kinase/protein kinase B
TGF-β: Transforming growth factor-beta
PDGF: Platelet-derived growth factor
EGFR: Epidermal growth factor receptor
TNF-α: Tumour Necrosis Factor-alpha
IL-1β: Interleukin-1 beta
CTGF: Connective tissue growth factor
GBM: Glioblastoma
MAPK: Mitogen-activated protein kinases
CKD: Chronic kidney disease
AKI: Acute kidney injury
DKD: Diabetic kidney disease
AT1R: Angiotensin II type 1 receptor
SOD: Superoxide dismutase
RA: Rheumatoid arthritis
EPA: Eicosapentaenoic acid
DHA: Docosahexaenoic acid
NF-κB: Nuclear factor kappa B
MT1-MMP: Membrane-type I matrix metalloproteinase
EMT: Epithelial-mesenchymal transition
ANP: Atrial natriuretic peptide
MAO: Monoamine oxidase
EGCG: Epigallocatechin gallate
α2M: α2-Macroglobulin
PTMs: Post-translational modifications
LC-MS: Liquid chromatography-mass spectrometry
gRNAs: guide RNAs
